# Thyroid transcriptome analysis reveals different adaptive responses to cold environmental conditions between two chicken breeds

**DOI:** 10.1371/journal.pone.0191096

**Published:** 2018-01-10

**Authors:** Shanshan Xie, Xukai Yang, Dehe Wang, Feng Zhu, Ning Yang, Zhuocheng Hou, Zhonghua Ning

**Affiliations:** National Engineering Laboratory for Animal Breeding and MOA Key Laboratory of Animal Genetics and Breeding, College of Animal Science and Technology, China Agricultural University, Beijing, China; Universitat de Lleida, SPAIN

## Abstract

Selection for cold tolerance in chickens is important for improving production performance and animal welfare. The identification of chicken breeds with higher cold tolerance and production performance will help to target candidates for the selection. The thyroid gland plays important roles in thermal adaptation, and its function is influenced by breed differences and transcriptional plasticity, both of which remain largely unknown in the chicken thyroid transcriptome. In this study, we subjected Bashang Long-tail (BS) and Rhode Island Red (RIR) chickens to either cold or warm environments for 21 weeks and investigated egg production performance, body weight changes, serum thyroid hormone concentrations, and thyroid gland transcriptome profiles. RIR chickens had higher egg production than BS chickens under warm conditions, but BS chickens produced more eggs than RIRs under cold conditions. Furthermore, BS chickens showed stable body weight gain under cold conditions while RIRs did not. These results suggested that BS breed is a preferable candidate for cold-tolerance selection and that the cold adaptability of RIRs should be improved in the future. BS chickens had higher serum thyroid hormone concentrations than RIRs under both environments. RNA-Seq generated 344.3 million paired-end reads from 16 sequencing libraries, and about 90% of the processed reads were concordantly mapped to the chicken reference genome. Differential expression analysis identified 46–1,211 genes in the respective comparisons. With regard to breed differences in the thyroid transcriptome, BS chickens showed higher cell replication and development, and immune response-related activity, while RIR chickens showed higher carbohydrate and protein metabolism activity. The cold environment reduced breed differences in the thyroid transcriptome compared with the warm environment. Transcriptional plasticity analysis revealed different adaptive responses in BS and RIR chickens to cope with the cold, and showed higher responsiveness in BS compared with RIR chickens, suggesting greater adaptability of the thyroid in BS chickens. Moreover, 10,053 differential splicing events were revealed among the groups, with RNA splicing and processing, gene expression, transport, and metabolism being the main affected biological processes, identifying a valuable alternative splicing repertoire for the chicken thyroid. A short isoform of *TPO* (encoding thyroid peroxidase) containing multiple open reading frames was generated in both breeds by skipping exons 4 and 5 in the cold environment. These findings provide novel clues for future studies of the molecular mechanisms underlying cold adaptation and/or acclimation in chickens.

## Introduction

Cold stress is one of the most prominent environmental challenges for homeothermic animals, with potentially negative effects on their reproduction, health, wellbeing, and feed efficiency [1–3]. Chickens are economically important animals that provide high-quality and affordable protein for human consumption. Currently, consumer demand for high-quality food from animals is coupled with an increasing emphasis on animal welfare [4], and free-range production systems are thus becoming more widespread, potentially subjecting the animals to more frequent and serious cold stress. Homeothermic animals protect themselves from the cold by increasing heat production and decreasing heat loss via a variety of physiological changes in multiple organs and systems [5]. Thyroid hormones (THs), including thyroxine (tetra-iodothyronine, T4) and tri-iodothyronine (T3), play important roles in energy metabolism and thermogenesis [6, 7]. THs are produced by the thyroid gland, which is organized into follicles filled with a secretory substance, colloid, and made up of cuboidal epithelial cells. Although investigations of avian thyroid function are less comprehensive than those on mammals, cold environments have been shown to enhance heat production and T3 concentrations in birds [8–11], dogs [12], and humans [13, 14]. Thyroid size and activity were also shown to increase in birds acclimated to low temperatures [15–17]. These findings suggest important roles for the thyroid in cold acclimation and/or adaptation.

Organisms can tolerate environmental changes via extensive transcriptomic rearrangements, while animal breeds originating from contrasting environments are well adapted to the local environments, indicating that both transcriptional plasticity and breed differences contribute to unique adaptability [18–21]. So far, numerous transcriptomic studies have investigated the mechanisms of heat tolerance in chickens [22–25], whereas cold tolerance is less well understood. Landmark heat-tolerance studies by Lamont and colleagues were performed using artificial temperature manipulations [22–25], but the difficulties in creating cold-stress conditions (< 0°C) for a large population (hundreds) of birds for a long period (a few months) make similar approaches unfeasible for cold-stress experiments. In contrast, natural winter environments provide a rational and convenient alternative solution.

The Bashang Long-tail (BS) is a Chinese indigenous breed of chicken found in the Bashang region in northern China (Hebei province, 41°14′—41°56′N, 114°50′—116°04′E; average altitude, 1418 m; mean annual temperature, 1.9°C). This breed is robust to the harsh local environment and is used as a dual-purpose breed for egg and meat production. Rhode Island Red (RIR) chickens were originally developed in Massachusetts and Rhode Island, USA, and are a famous dual-purpose breed characterized by hardiness and good performance (The American Livestock Breeds Conservancy, http://albc-usa.org/cpl/rhodered.html). We previously showed that RIRs performed better under warm environments, while BS and reciprocal BS/RIR crossbreds exhibited higher performances under cold environments, indicating the higher cold-tolerance of BS chickens and the feasibility of simultaneously improving cold tolerance and performance [26]. In the current study, we investigated the egg production performance, body weight changes, serum TH concentrations, and thyroid gland transcriptome profiles of BS and RIR chickens under cold and warm environments. We aimed to compare the cold tolerances of BS and RIR birds, and explore breed differences in the thyroid transcriptome and thyroid transcriptomic cold-induced plasticity. Derived information will deepen our understanding of the regulatory mechanisms underlying cold tolerance.

## Materials and methods

### Ethics statement

The animal care protocol used in the present study was approved by the Animal Welfare Committee of China Agricultural University (permit number: DK996).

### Animals and treatments

BS and RIR chickens were purchased from the Lvtianyuan chicken farm in Bashang region and the Beijing Zhongnong Bangyang Layer Breeding Co., Ltd. (Beijing), respectively. The animals and treatments used in the present study have been described previously [26]. Briefly, cold-stress birds (n = 102, 18-week-old females) were randomly assigned to six sheds maintained under the natural cold environment (34 birds/shed, 0.17m^2^/bird, 3 sheds/breed), and the control birds (n = 102 per BS/RIR, 18-week-old females) were caged in a laying house maintained under warm conditions. All the birds were given *ad libitum* access to clean water and feed (metabolizable energy, 2.60 Mcal/kg; crude protein, 15.20%). A 16 h light/1 h dark cycle was maintained until the end of the experiment (39 weeks). Daily maximum/minimum temperatures were recorded and daily mean temperature was expressed as the average of the daily maximum/minimum temperatures. The temperature ranges for the cold-stress and control birds during the entire period (18 to 39 weeks) were −17.5°C to 27.0°C and 7.4°C to 26.5°C, respectively. From 30 to 39 weeks, the temperatures for the cold-stress birds fell below 0°C (< −5°C from 37 weeks), compared with around 10°C for the control birds. Bird mortality was low in both environments throughout the entire experimental period (warm environment: BS 1, RIR 2; cold environment: BS 3, RIR 3).

### Parameters and statistical analysis

Egg production performance and body weight were monitored as described previously [26]. Briefly, the weekly laying rate was calculated as follows:

$$Weekly\;laying\;rate=\frac{Total\;number\;of\;eggs\;produced\;during\;7\;days}{Total\;number\;of\;hen-days\;in\;the\;same\;period}$$ (1)

Blood samples were collected from the birds at 39 weeks of age, after long-term cold stress (< 0°C for about 10 weeks), when the temperatures for the cold-stress and control birds were about −10°C and 13°C, respectively. The blood samples (3 mL) were collected from the brachial vein into vacuum blood collection tubes (n = 20 birds/breed·environment) and kept in an incubator (37°C) overnight for clotting. The clot was then removed by centrifugation and the resulting supernatant (serum) was carefully collected. Serum T3 and T4 levels were measured using commercially available ^125^I-labeled radioimmunoassay kits (Sino-UK institute of Biological Technology, Beijing, China).

Body weight data were analyzed with two-way ANOVA using the general linear model procedure (GLM, SPSS for Windows Release 20.0, SPSS Inc.). The main effects of temperature, genotype, and their interaction were tested. If a significant interaction of the main effects were detected, then the simple main effects were tested using the syntax of SPSS to determine the mean difference between breeds under the same environment, as well as between environments within the same breed. Serum TH concentrations were analyzed with two-way MANOVA to detect the overall effects of breed and environment on the hormone levels. Post-hoc univariate ANOVA was then performed to test the effects of breed, environment, and their interaction on the hormone levels. Simple main effects analysis was also performed. Significance was set at *p* < 0.05.

### Thyroid sampling, RNA extraction and sequencing

Thyroid sampling was performed immediately after blood sampling (4 biological replicates, 16 samples in total). Avian thyroid glands are located ventrolateral to the trachea, just caudal to the junction of the subclavian and common carotid arteries [15]. The birds were euthanized by cervical dislocation to alleviate pain and suffering, and thyroid gland samples were then excised and immediately frozen in liquid nitrogen. Total RNA was extracted from the thyroid samples with TRIzol reagent (Invitrogen) according to manufacturer’s instructions. RNA quality and concentrations were assessed with an Agilent 2100 Bioanalyzer. The RNA integrity number (RIN) threshold was 7.5 for library construction. mRNA was purified from total RNA using poly-T oligo-attached magnetic beads. Finally, 16 libraries with 250–350 bp insert sizes were prepared using the TruSeq RNA Sample Prep Kit v2 (Illumina, San Diego, CA, USA). Illumina HiSeq4000 (PE150) and HiSeq 2500 (PE125) platforms were employed to obtain transcriptome profiles. The raw sequence reads have been deposited in the NCBI sequence read archive (SRA PRJNA357330). The RNA samples were also reserved for quantitative real-time PCR (qPCR) analysis.

### Whole-transcriptome sequencing data processing and differential gene expression analysis

Read quality was evaluated using the FastQC suite version 0.10.1 (http://www.bioinformatics.babraham.ac.uk/projects/fastqc/), and quality trimming was performed using Trimmomatic-0.36 [27] with parameters set as HEADCROP:19 SLIDINGWINDOW:4:15 MINLEN:60. After quality filtering, paired reads were extracted and mapped to the ENSEMBL genome build Gallus_gallus-5.0 (GCA_000002315.3) using HISAT2 version 2.0.5 [28]. StringTie version 1.3.3b [29, 30] was used to assemble the alignments into potential transcripts. Sorting and indexing of the read alignment files were implemented with SAMtools version 1.4 [31]. Mapping statistics were calculated with SAMtools and RSeQC (version 2.6.3) [32]. We compared the assembled transcripts to the reference genome using gffcompare version 0.10.1 (available from http://ccb.jhu.edu/software/stringtie/gff.shtml). Transcripts coded with “u”, “i”, or “x” were recognized as new loci, and those coded with “j”, “o”, or “e” were treated as new transcripts of known loci from the annotation database.

A Python script (prepDE.py) provided by the StringTie maintainer (available at https://ccb.jhu.edu/software/stringtie/dl/prepDE.py) was used to generate a gene count matrix based on the StringTie outputs. The R (version 3.4.0) [33] package edgeR version 3.18.0 [34] was used to detect differentially expressed genes (DEGs) using TMM normalization and quasi-likelihood (QL) F-test, which provides a more robust and reliable error rate control [35, 36]. Raw gene counts were filtered with counts per million (cpm > 2) to eliminate genes with low counts across multiple samples (< 3 samples), and 13,678 genes passed the filtering. Multidimensional scaling (MDS) analysis was performed using the function plotMDS within edgeR package to investigate the overall gene expression patterns, and 3 samples (BS_Cold2, RIR_Cold1, and BS_Warm4) recognized as outliers were removed from differential gene expression analysis. DEGs were identified at FDR < 0.05 (Benjamini-Hochberg correction [37]) and absolute fold change ≥ 1.5 (|log2FC| ≥ 0.58). Venn diagrams were created using the online tool Venny 2.1.0 (http://bioinfogp.cnb.csic.es/tools/venny/index.html). FPKM (fragments per kilobase million) values of gene expression were also extracted from the StringTie outputs. R studio (https://www.rstudio.com/) was used to run custom scripts to construct heat maps, box plots, and density plots. Unless otherwise stated above, all the programs were run with the default parameter settings.

### GO and KEGG pathway analysis

Functional classification of DEGs was performed using the Panther Classification System (Version 12.0) [38] and WebGestalt [39] (data source updated on 1/27/2017). GO enrichment analysis was performed using the statistical enrichment test tool within Panther (GO Ontology database released 2017-06-29), which takes full gene lists ranked by gene expression fold changes as input to determine whether any ontology category or pathway has numeric values that are nonrandomly distributed with respect to the entire list of values using the Mann-Whitney rank-sum test [38]. This method considers a large number of genes with weaker expression changes but which may have important effects, providing increased statistical power to reveal the underlying mechanisms behind global gene expression changes [40]. KEGG pathway enrichment analysis was performed using the gene set enrichment analysis (GSEA) within WebGestalt [39], with 1000 permutations and using Benjamini-Hochberg multiple testing correction [37]. This method also analyzes a ranked full gene list to maximize information usage. Unless otherwise stated above, all the programs were run with the default parameters.

### Differential splicing analysis

RNA-Seq has the advantage of enabling the detection of alternative splicing events between different conditions. We analyzed differential splicing among the groups using DEXSeq [41], which adopts the exon-based approach to detect signals of differential splicing by comparing the distributions of reads on exons and junctions (exon usage) of genes between the compared samples. DEXSeq uses generalized linear models (GLMs) to perform statistical analysis, and reports fold changes and corrected *p* values for differential usage of each exon. Differential splicing events were determined at Benjamini-Hochberg adjusted *p*-value < 0.05. GO and KEGG pathway overrepresentation analysis for the differentially spliced genes was performed using the Panther Classification System [38] (Statistical overrepresentation test) and WebGestalt [39] (overrepresentation enrichment analysis; data source updated on 1/27/2017), respectively.

### Quantitative real-time PCR (qPCR) validation

The expression levels of 10 DEGs were tested by qPCR with the RNA samples used for RNA-Seq. First-strand cDNA synthesis was performed using an EasyScript cDNA Synthesis Super Mix kit (TransGen Biotech, Beijing, China). Specific primers were designed using primer3 [42], and were aligned against the chicken genome using the NCBI Primer-BLAST tool to avoid multiple amplification [43]. The primers were designed to span the exon/intron boundaries to avoid amplification of potential residual genomic DNA. All qPCR analyses were performed in triplicate on the ABI Prism 7500 sequence detection system (Applied Biosystems group) using SYBR green chemistry. The qPCR was performed as follows: denaturation at 95°C for 5min followed by 40 cycles (95°C for 10s, 60°C for 1 min). Relative gene expression levels were calculated using the 2^-ΔΔCt^ method, with glyceraldehyde 3-phosphate dehydrogenase (*GAPDH*) as an internal control.

## Results and discussion

### Egg production performance and body weight

**Fig 1:**
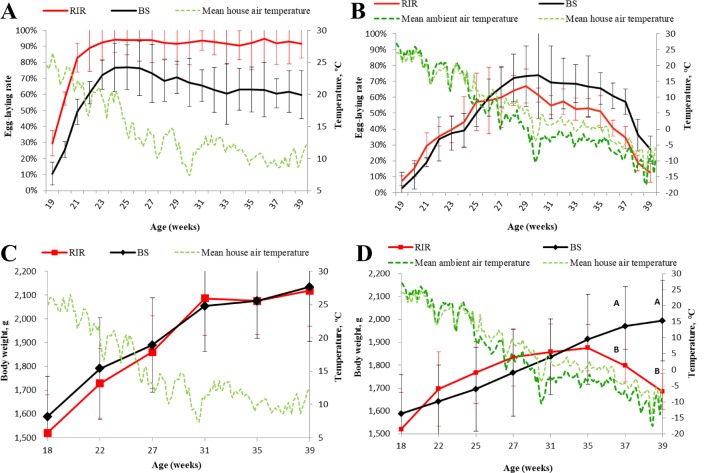
Weekly laying rates and body weights of BS and RIR chickens in warm and cold environments. (A, C) Weekly laying rates and body weights of the two breeds in warm environment. (B, D) Weekly laying rates and body weights of the two breeds in cold environment. Values are given as mean ± SD. At a given age in D, different letters denote a significant difference (*p* < 0.01).

Chickens can only tolerate ambient temperature fluctuations within certain ranges, and suboptimal conditions have negative impacts on egg-laying performance and body weight [44–46]. The temperature in the cold environment was < 0°C from 30 to 39 weeks, and was < −5°C after 37 weeks, representing severe cold stress compared with the temperatures experienced by the control birds (around 10°C; Fig 1). The peak laying rates of the RIR and BS control birds during the entire experimental period were 95.0% and 77.0% (χ2 = 13.4, *p* = 2.5×10^−4^, Fig 1A), respectively, compared with 67.0% and 74.0% for the equivalent cold-stress birds (χ2 = 1.1, *p* = 0.29; Fig 1B). The egg production performance from 18–39 weeks was higher in RIR compared with BS birds in the warm environment (130.7 vs. 91.1 eggs/bird, *p* < 1.0×10^−7^), while BS birds produced more eggs than RIR birds in the cold environment (70.5 vs. 57.7 eggs/bird, *p* = 5.2×10^−4^). Reproduction is an energy-intensive process during which a large amount of nutrients are transferred from the body to the egg. A reasonable response to cold stress is thus to reallocate internal resources from reproduction to other fate-determining processes, such as maintaining a stable body temperature [47, 48]. Moreover, extended cold stress is likely to induce body weight decreases because of the long duration of enhanced catabolism. We compared the body weights of chickens maintained in cold and warm environments (Fig 1D to Fig 1C) and showed that the cold stress repressed the body weight increases in both breeds; however, body weight decreased at the late stage in RIR but not in BS birds, reflecting more serious negative impacts of cold exposure on RIR chickens. These results suggest better cold tolerance of BS chickens, which thus are preferred for selection for cold tolerance.

### Serum TH concentrations

T3 and T4 are produced by the thyroid gland and are primarily responsible for the regulation of metabolism. Considerable evidence suggests that thyroid gland activity and/or serum TH concentrations increase when birds become acclimated to cold environments [8, 9, 15–17]. Previous studies [10–12, 49–51] have also demonstrated that animals living in cold regions have higher T3 and/or T4 levels than their counterparts living in warmer regions. Similar results were found in the present study. Serum T3 and T4 concentrations were measured at 39 weeks (Table 1), when the temperatures were about −10°C and 13°C for the cold-stress and control birds (Fig 1), respectively. T3 and T4 levels tended to increase in both breeds in the cold compared with the warm environment, suggesting an increased metabolic rate [9, 52]. It is noteworthy that BS chickens had significantly higher TH levels than RIR chickens under both environments (*p* < 0.05). BS chickens originated from a relatively cold region with an average altitude of 1418 m and a mean annual temperature of 1.9°C, suggesting that higher TH levels may be a potential signature of cold tolerance. Genotype and environment interactions (G×E) had a significant influence on T4 concentrations (*p* < 0.05).

**Table 1:** Serum thyroid hormone concentrations in two chicken breeds in cold and warm environments at 39 weeks (n = 20, Mean ± SD).

Item	Cold		Warm		*p*-value		
	BS	RIR	BS	RIR	E	G	G×E
T3	0.72±0.15^a^^x^	0.60±0.15^b^^x^	0.68±0.13^a^^x^	0.55±0.13^b^^x^	0.15	0.001	0.92
T4	33.56±8.78^a^^x^	25.69±10.58^b^^x^	28.03±10.46^a^^y^	19.60±7.98^b^^x^	0.01	0.90	0.001

^a, b^ Different superscript letters indicate significant difference between breeds under the same environment (*p* < 0.05).

^x, y^ Different superscript letters indicate significant difference between environments within the same breed (*p* < 0.05).

E: environment; G: genotype; G×E: genotype and environment interactions.

### Transcriptome overview

**Fig 2:**
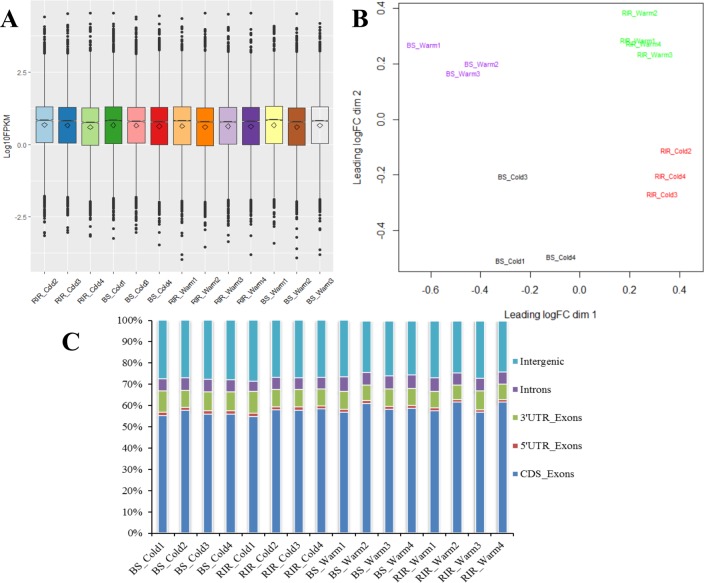
Gene expression patterns and mapped read distributions. (A) Distributions of gene expression levels. Each box plot contains hinges at the 25^th^ and 75^th^ percentile, a line at the median, and a rhombus at the mean. (B) MDS plots showing the divergence between groups. (C) Distributions of the mapped reads.

RNA-Seq yielded around 344.3 million paired-end reads. Detailed information on data filtering and mapping statistics are presented in Table 2. For the cold-stress birds (8 samples), an average of 21.1 million paired-end reads per sample passed the quality trimming, resulting in a concordant mapping rate of 89.46%–91.17%. For the control birds (8 samples), an average of 19.6 million paired-end reads per sample passed the quality trimming, resulting in a concordant mapping rate of 88.46%–90.05%. Among the samples, splice reads accounted for 31.06%–38.75%. For each of the samples, 16,768–18,333 genes were detected (read counts > 0), accounting for 67.39%–73.68% of the 24,881 genes annotated in the reference genome (GCA_000002315.3). Across the samples, a total of 21,303 expressed genes were detected (read counts > 0), accounting for 85.60% of the total annotated genes. To further examine the read distribution, the mapped reads (excluding QC fail, duplicate and non-primary hit reads) were assigned to exon coding region (CDS_Exons), 5′ untranslated regions (5′ UTR_Exons), 3′ untranslated regions (3′ UTR_Exons), introns, and intergenic regions (Fig 2C). Most of the reads (54.66%–61.42%) were aligned to CDS_Exons, 1.17%–1.53% were mapped to 5′ UTR_Exons, 6.83%–10.29% were mapped to 3′ UTR_Exons, and only 4.90%–6.95% were mapped to introns. Similar to the findings of Yi et al. [53], a relatively large proportion of the reads (24.37%–28.61%) were mapped to intergenic regions, indicating the need to improve the chicken reference annotation [54]. Comparisons between the assembled transcripts and the reference genome were performed using gffcompare. Depending on the group of birds, 59,969–63,842 transcripts were assembled, of which 58.99%–62.80% were predicted to have exactly the same introns as the reference transcript. Moreover, 23.20%–26.27% were recognized as potential novel isoforms of known loci, and 11.14%–14.73% were from new loci (S1 Table), attesting to the power of RNA-Seq to discover novel transcripts.

**Table 2:** Statistics for the filtering and mapping of reads.

Sample	Total reads (M)	Filtered reads (M)	Mapped reads %	Uniquely mapped reads %^a^	Concordantly mapped reads %^a^	Splice reads %^a^	Genes with counts	Transcriptome %^b^
BS_Cold_1	21.87	20.70	86.02%	95.57%	89.87%	31.25%	17634	70.87%
BS_Cold_2*	22.82	20.67	89.38%	95.88%	89.79%	32.67%	17172	69.02%
BS_Cold_3	20.56	18.59	87.89%	95.82%	89.46%	31.60%	17465	70.19%
BS_Cold_4	22.58	20.35	90.44%	95.80%	89.97%	31.47%	17575	70.64%
RIR_Cold_1*	20.96	19.11	88.15%	95.92%	90.44%	31.06%	17257	69.36%
RIR_Cold_2	19.62	17.96	91.50%	95.92%	91.12%	32.45%	16898	67.92%
RIR_Cold_3	16.87	15.41	91.50%	96.11%	91.17%	32.39%	16768	67.39%
RIR_Cold_4	23.62	21.61	91.43%	95.93%	91.09%	32.91%	17432	70.06%
BS_Warm_1	21.29	18.84	89.99%	95.71%	89.13%	34.59%	18283	73.48%
BS_Warm_2	22.75	20.84	90.64%	95.54%	88.78%	38.08%	18333	73.68%
BS_Warm_3	23.11	21.07	89.93%	95.64%	88.46%	36.39%	18301	73.55%
BS_Warm_4*	22.29	19.77	91.09%	95.76%	88.85%	36.07%	17731	71.26%
RIR_Warm_1	22.22	19.59	90.73%	95.99%	89.18%	35.40%	17451	70.14%
RIR_Warm_2	22.72	20.54	90.87%	95.83%	90.05%	38.75%	17513	70.39%
RIR_Warm_3	20.84	18.44	90.84%	95.95%	89.28%	35.07%	17280	69.45%
RIR_Warm_4	20.19	18.01	91.22%	95.87%	89.43%	38.53%	17244	69.31%

^a^ Uniquely mapped reads %, concordantly mapped reads %, and splice reads % were calculated based on the total mapped reads.

^b^ Transcriptome % was calculated using the number of detected genes divide the number of annotated genes in the reference genome.

* Indicates samples recognized as outliers by multidimensional scaling (MDS) analysis.

Investigations of highly expressed genes can provide insights into tissue-specific transcriptome characteristics; however, transcriptomic studies of the chicken thyroid gland are currently very limited. Here we investigated the top 200 highly expressed genes in each group (S1 File) and their functional distributions. The top 200 genes in different groups were similar, with 167 genes shared among the groups, and a total of 245 genes across the groups (S5 Fig). The functional distributions were also similar (S1–S4 Figs). KEGG pathway analysis was performed for the 245 highly expressed genes using WebGestalt [39]. Many genes were related to “ribosome”, “oxidative phosphorylation”, “lysosome”, “phagosome”, “glycolysis/gluconeogenesis”, and “tyrosine metabolism” (Table 3), all of which are involved in TH biosynthesis and release [55]. Thyroglobulin (*TG*), which is one of the key elements in TH synthesis [56], was the most highly expressed gene in every group. Thyroid peroxidase (*TPO*), which oxidizes iodide ions to iodine atoms for the production of THs [57], was also highly expressed in each group. Iodotyrosine deiodinase (*IYD*), which is involved in iodide salvage for iodide recycling [58, 59], was also within the top 200 genes in each group. All these results were in accord with the findings for the thyroid in humans [60] and pigs [61].

**Table 3:** KEGG pathway analysis for the overall top 245 highly expressed genes^#^.

Gene set	Description	#Genes	% of top 245 genes
gga03010	ribosome	63	25.71%
gga00190	oxidative phosphorylation	15	6.12%
gga04142	lysosome	9	3.67%
gga04141	protein processing in endoplasmic reticulum	10	4.08%
gga04145	phagosome	9	3.67%
gga00010	glycolysis/gluconeogenesis	5	2.04%
gga04520	adherens junction	4	1.63%
gga04514	cell adhesion molecules (CAMs)	5	2.04%
gga03320	PPAR signaling pathway	3	1.22%
gga01230	biosynthesis of amino acids	3	1.22%
gga00350	tyrosine metabolism	2	0.82%

^#^ Top 200 highly expressed genes in each group totaled 245 genes across groups.

### Differentially expressed genes and functional overview

**Fig 3:**
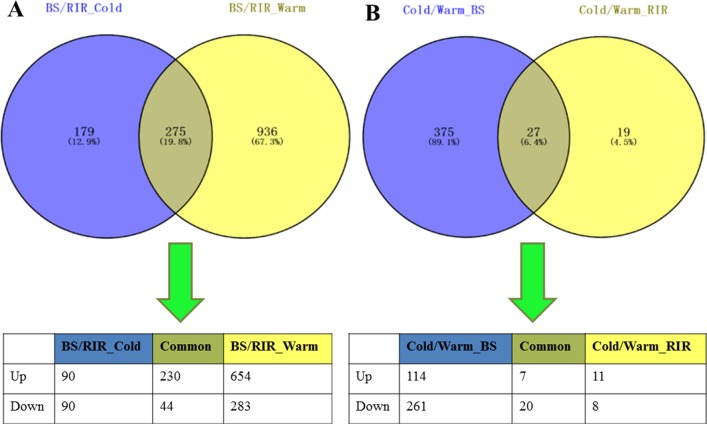
Venn diagrams. (A) Venn diagram for DEGs of BS/RIR_Cold and BS/RIR_Warm. One common significant gene, which was regulated at different directions in the two comparisons, was counted in the respective comparisons (bottom panel). (B) Venn diagram for DEGs of Cold/Warm_RIR and Cold/Warm_BS.

**Fig 4:**
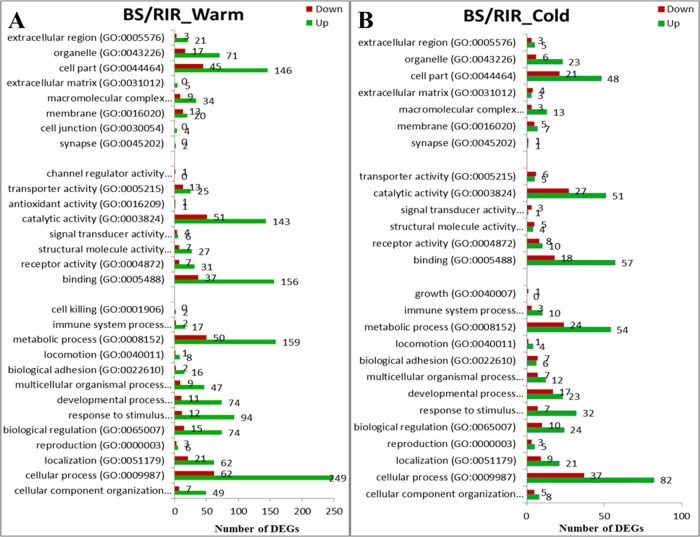
Functional classifications of up- and down-regulated genes for BS/RIR_Warm and BS/RIR_Cold.

**Fig 5:**
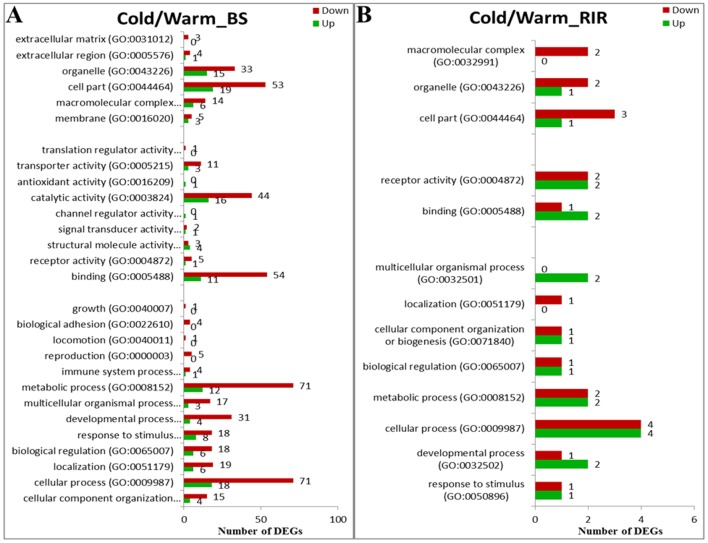
Functional classifications of up- and down-regulated genes for Cold/Warm_BS and Cold/Warm_RIR.

Prior to differential expression analysis, the gene count data were filtered for counts per million (cpm > 2) to eliminate genes with low counts across multiple samples (< 3 samples), resulting in a final count matrix with 13,478 genes. MDS analysis was performed to characterize the overall effects of cold stress and breed on gene expression, and 3 samples (BS_Cold2, RIR_Cold1, and BS_Warm4) recognized as outliers (S6 Fig) were removed from the differential gene expression analysis. Boxplots revealed similar distributions of gene expression levels among samples (Fig 2A). MDS analysis showed that the biological replicates of different groups were separable (Fig 2B), reflecting the effects of both internal (genetic background) and external factors (environmental effects) on gene expression. DEGs between breeds within the same environment (BS/RIR_Cold and BS/RIR_Warm) and DEGs between environments within the same breed (Cold/Warm_RIR and Cold/Warm_BS) were detected and summarized in Table 4 and Fig 3 (for the full DEGs list, see S2–S5 Files). GO functional annotations of the DEGs (Figs 4 and 5) were explored using the Panther system.

**Table 4:** Differentially expressed genes.

Comparison	Absolute mean fold change, all genes^b^	Mean fold change, DEGs	Total DEGs	Up-regulated	Down-regulated
BS/RIR^a^_Cold	0.48	1.68	454	320 (70.5%)	134 (29.5%)
BS/RIR^a^_Warm	0.49	1.70	1211	884 (73.0%)	327 (27.0%)
Cold/Warm^a^_RIR	0.36	2.60	46	18 (39.1%)	28 (60.9%)
Cold/Warm^a^_BS	0.45	1.52	402	121 (30.1%)	281 (69.9%)

^a^ Set as control group in each comparison. Sample sizes: BS_Cold, 3; BS_Warm, 3; RIR_Cold, 3; RIR_Warm, 4.

^b^ Gene expression data were fed into edgeR and filtered using the function rowSums(cpm(y)>2)≥3.

There were significantly more DEGs between breeds under the warm compared with the cold environment (Fig 3A, Table 4; 1211 vs. 454 χ^2^ = 365.86, *p <* 2.20×10^−16^). For BS/RIR under both environments, the numbers of up-regulated genes were significantly greater than the numbers of down-regulated genes (Table 4; BS/RIR_Cold, 320 vs. 134, χ^2^ = 75.39, *p <* 2.20×10^−16^; BS/RIR_Warm, 884 vs. 327, χ^2^ = 255.27, *p <* 2.20×10^−16^). There were more extensive differences in the related physiological processes under the warm environment (Fig 4A and 4B). In both BS/RIR_Cold and BS/RIR_Warm, most of the up-regulated DEGs were assigned to “metabolic processes”, “cellular processes”, “binding”, and “catalytic activity” (Fig 4A and 4B). Closer investigation of the up- and down-regulated genes in BS/RIR_Cold and BS/RIR_Warm showed that 274 of the 275 common DEGs were regulated in the same direction (Fig 3A), and only one gene (glycerol-3-phosphate dehydrogenase 1 like, *GPD1L*) was regulated in the opposite directions, being up- and down-regulated in BS/RIR_Warm and BS/RIR_Cold, respectively. Further analysis showed that *GPD1L* was down-regulated in Cold/Warm_BS (log2FC = –0.96, FDR = 0.015) and up-regulated in Cold/Warm_RIR (log2FC = 0.32, FDR = 0.41). The GPD1L protein catalyzes the conversion of sn-glycerol 3-phosphate to glycerone phosphate [62], and decreased enzymatic activity of protein GPD1L induces increased levels of glycerol 3-phosphate, which activate the GPD1L-dependent cardiac sodium channel (Na_V_1.5) phosphorylation pathway, leading to a decrease in the sodium current in the heart [63, 64]. Previous co-expression experiments also showed that mutant GPD1L reduced the sodium current in HEK293 cells [64]. However, the functions of GPD1L in the thyroid remain unclear.

Most of the DEGs in BS/RIR_Cold (274 of 454 DEGs, 60.35%, Fig 3A) were subsets of the BS/RIR_Warm DEGs, suggesting that those DEGs reflecting breed differences were robust to external thermal environmental variation. A total of 937 of 1,211 DEGs (77.37%) in BS/RIR_Warm were eliminated in BS/RIR_Cold, while only 180 other DEGs (90 up-regulated, 90 down-regulated) were introduced (Fig 3A), indicating that the cold environment generally repressed transcriptome differences between the breeds.

We identified 402 and 46 DEGs in Cold/Warm_BS and Cold/Warm_RIR, respectively (Table 4, Fig 3B). Most of the DEGs in Cold/Warm_BS were down-regulated (121 up-regulated vs. 281 down-regulated genes, χ^2^ = 62.89, *p = 2*.19×10^−15^), and related to “metabolic processes”, “cellular processes”, “binding”, and “catalytic activity” (Fig 5A), which was in accord with the decreased breed differences in these categories in BS/RIR_Cold compared with BS/RIR_Warm (Fig 4A and 4B). Meanwhile, only 46 DEGs were detected in Cold/Warm_RIR (18 up-regulated vs. 28 down-regulated genes, χ^2^ = 1.76, *p =* 0.18; Table 4, Fig 3B), which was significantly fewer than in Cold/Warm_BS (46 vs. 402, χ^2^ = 286.06, *p <* 2.20×10^−16^; Table 4), suggesting greater adaptability of the thyroid in BS chickens. The higher responsiveness of BS chickens may reflect a higher robustness and potential to acclimate and survive cold temperatures, supported by the higher egg production performance and more stable body weight gain in BS compared with RIR chickens under the cold (Fig 1). Windisch et al. also find a positive relationship between gene expression responsiveness and overall survivability in fish challenged by cold [65]. The interaction between environment and breed did not show significant effects on gene expression.

### Breed differences in thyroid transcriptome profiles under cold and warm environments

Genetic background defines the adaptability of organisms, and comparing breed differences in thyroid transcriptome profiles thus allow us to gain insight into the breed-specific molecular basis of the adaptability. In addition to the significant DEGs that passed the multiple testing correction, a typical digital gene expression study will generally identify abundant genes with weaker expression changes, which are generally ignored by traditional functional enrichment analyses (based on the hypergeometric, chi-square, or binomial distribution) and which are thus ignored by the interpretation of phenotype changes. However, these abundant, less-changed genes may also have significant effects [40, 66]. To make full use of the information of the less-changed genes in the study, we used the statistical enrichment test tool in the Panther to reveal the significantly enriched GO categories behind the global gene expression changes. There were many more significantly enriched GO terms in BS/RIR_Warm than in BS/RIR_Cold (Table 5), indicating larger breed differences in the thyroid transcriptome profiles under warm compared with cold conditions, supported by the more DEGs in BS/RIR_Warm (Table 4). Most of the enriched categories (14/16) in BS/RIR_Warm were overrepresented, as well as all the categories in BS/RIR_Cold, reflecting the tendency for the related genes to be highly expressed in BS chickens, which may contribute to higher serum TH concentrations (Table 1). “Immune response” and “DNA replication” were overrepresented in both BS/RIR_Warm and BS/RIR_Cold, reflecting that these differences between breeds were robust to external temperature environments (Table 5). The overrepresentations of “developmental process”, “system development”, “DNA replication”, “DNA metabolic process”, “cell cycle”, “regulation of transcription from RNA polymerase II promoter”, “nucleobase-containing compound metabolic process”, and “cell differentiation” in BS/RIR_Warm suggested higher cell replication and development activity in the BS thyroid. “Cytokine-mediated signaling pathway” was also overrepresented in BS/RIR_Warm. Only two categories, “fatty acid beta-oxidation” and “carbohydrate metabolic process”, were underrepresented in BS/RIR_Warm.

**Table 5:** GO enrichments for BS/RIR under cold and warm environments.

Biological process	#Genes	BS/RIR_Warm		BS/RIR_Cold	
		+/−^a^	*p* value^b^	+/−^a^	*p* value^b^
immune response (GO:0006955)	161	+	1.82E-10	+	1.77E-04
regulation of nucleobase-containing compound metabolic process (GO:0019219)	425	+	3.94E-09		
response to stimulus (GO:0050896)	787	+	2.24E-08		
nucleobase-containing compound metabolic process (GO:0006139)	1160	+	1.25E-07		
regulation of transcription from RNA polymerase II promoter (GO:0006357)	245	+	7.08E-07		
developmental process (GO:0032502)	607	+	3.72E-06		
system development (GO:0048731)	326	+	3.50E-05		
DNA replication (GO:0006260)	52	+	4.52E-05	+	2.33E-02
DNA metabolic process (GO:0006259)	151	+	1.19E-03		
cell cycle (GO:0007049)	364	+	2.15E-03		
response to interferon-gamma (GO:0034341)	19	+	3.43E-03		
fatty acid beta-oxidation (GO:0006635)	19	−	7.52E-03		
carbohydrate metabolic process (GO:0005975)	211	−	8.53E-03		
cellular defense response (GO:0006968)	66	+	1.41E-02		
cytokine-mediated signaling pathway (GO:0019221)	40	+	1.72E-02		
cell differentiation (GO:0030154)	133	+	2.05E-02		

^a^ +/− Overrepresentation or underrepresentation.

^b^ Bonferroni-corrected *p* value.

In the KEGG pathway enrichment analysis, all the enriched KEGG pathways were up-regulated in RIR chickens (ES and NES < 0; Table 6). Only one pathway was returned in BS/RIR_Cold, while more pathways were returned in BS/RIR_Warm. Under both conditions, the “citrate cycle (TCA cycle)” pathway, which is an important aerobic pathway for the oxidation of carbohydrates and fatty acids [67], was up-regulated in RIR, suggesting higher metabolic activity in the thyroid of RIR birds. Other carbohydrate metabolism-related pathways such as “propanoate metabolism”, “glyoxylate and dicarboxylate metabolism”, and “butanoate metabolism” were also up-regulated in RIR under the warm environment, in accordance with the underrepresentations of the metabolism-related categories (e.g., “fatty acid beta-oxidation”, and “carbohydrate metabolic process”) in BS under the warm environment (Table 5). Higher protein metabolism-related activity (“valine, leucine and isoleucine degradation”, “proteasome”, “protein export”, “ribosome”, and “aminoacyl-tRNA biosynthesis”) was also revealed in RIR chickens under warm conditions.

**Table 6:** GSEA tests for KEGG pathway enrichments for BS/RIR under cold and warm environments.

Gene set	Description	BS/RIR_Warm			BS/RIR_Cold		
		ES	NES	FDR	ES	NES	FDR^a^
gga00020	citrate cycle (TCA cycle)	−0.392	−2.460	0.000	−0.302	−1.838	0.092
gga00280	valine, leucine and isoleucine degradation	−0.302	−2.583	0.000			
gga03010	ribosome	−0.261	−3.839	0.000			
gga03050	proteasome	−0.392	−2.922	0.000			
gga03060	protein export	−0.431	−2.387	0.001			
gga00640	propanoate metabolism	−0.333	−2.227	0.003			
gga00630	glyoxylate and dicarboxylate metabolism	−0.366	−2.207	0.003			
gga00970	aminoacyl-tRNA biosynthesis	−0.289	−1.899	0.030			
gga00790	folate biosynthesis	−0.451	−1.833	0.037			
gga00650	butanoate metabolism	−0.359	−1.843	0.038			

^a^ BH-adjusted *p* value.

Taken together, the results of GO and KEGG enrichment analyses indicated that the BS thyroid was characterized by higher cell replication and development, and immune response-related activity, while higher carbohydrate and protein metabolism activity were revealed in the RIR thyroid. A total of 26 and 3 enrichments (GO plus KEGG) were identified in warm and cold conditions, respectively, also indicating that the cold reduced the breed differences in the thyroid transcriptome, in accord with the results of DEGs.

Given the observation that serum TH concentrations were higher in BS chickens under both environments (Table 1), we investigated the expression levels of the genes involved in TH biosynthesis, secretion, and transportation. Based on the thyroid-related genes reported in humans [60] and pigs [61], we obtained the chicken orthologues (12 genes, S6 File) using BioMart [68]. Among the 12 thyroid-related genes, *GPX3*, *TPO*, and *TG* were extremely highly expressed, with FPKM of 591–31246, while *ADCY1*, *DDC*, and *DIO2* were extremely lowly expressed, with FPKM of 0.0–1.96. These findings were similar to the scenario in humans [60]. With respect to breed differences, only one gene, *TSHR*, was found to be differentially expressed, and was up-regulated in RIR compared with BS chickens under cold conditions (FDR < 0.05). *TSHR* encodes the thyroid-stimulating hormone receptor that stimulates the follicular cells to produce T4 and T3 [69]. Thyroid gland activity is controlled by the hypothalamus–pituitary–thyroid axis; the downstream organs sense and respond to the signals conveyed by various hormones produced by the upstream organs, while induced hormones in the downstream organs also provide negative feedback to decrease levels of the hormones stimulating their release [70]. TH biosynthesis and release comprise a complex biological process involving a group of genes and multiple steps, including iodide ion concentration and oxidation, iodine organification, coupling of monoiodotyrosine and diiodotyrosine, and TH storage and release [71, 72]. In the body, serum T3 and T4 levels are in a state of dynamic balance, affected on the one hand by the synthesis and secretion rates of the thyroid gland, and on the other hand by the metabolism and clearance rates in peripheral tissues [73, 74]. Further comprehensive studies are needed to clarify the regulatory mechanisms that determine serum TH concentrations.

### Transcriptional plasticity in the thyroid

Exposure of organisms to suboptimal conditions may induce appropriate responses to maximize fitness. Transcriptional plasticity is defined as the capacity for a gene to change its transcriptional level under different conditions, which contributes to phenotypic diversity and adaptability [19, 75]. We analyzed the transcriptional plasticity of the thyroid in BS and RIR chickens to characterize the molecular mechanisms underpinning cold acclimation, which remains largely unexplored in homeothermic animals.

Table 7 shows the GO enrichments for global gene expression plasticity. Many more GO terms were enriched in Cold/Warm_BS (14 terms) compared with Cold/Warm_RIR (2 terms), indicating greater adaptability in BS chickens, supported by the more DEGs in Cold/Warm_BS. In Cold/Warm_RIR, two RIR-specific enrichments (“response to stimulus” and “immune response”) were detected, and both of them were overrepresented (tend to be up-regulated in the cold); while most of the enrichments (13/14) in Cold/Warm_BS were underrepresented (tend to be down-regulated in the cold). BS-specific responses to the cold included the underrepresentations of “developmental process”, “system development”, “cell differentiation”, “regulation of transcription from RNA polymerase II promoter”, “nucleobase-containing compound metabolic process”, and “regulation of nucleobase-containing compound metabolic process”, these results supported the finding that the same biological processes were overrepresented in BS/RIR_Warm, but eliminated in BS/RIR_Cold. Other BS-specific responses were the underrepresentations of many biological processes involved in signal transduction, such as “signal transduction”, “cell communication”, and “intracellular signal transduction”.

**Table 7:** GO enrichments for gene expression plasticity in BS and RIR chicken breeds.

Biological process	#Genes	Cold/Warm_BS		Cold/Warm_RIR	
		+/−^a^	*p* value^b^	+/−^a^	*p* value^b^
regulation of nucleobase-containing compound metabolic process (GO:0019219)	425	−	9.09E−08		
signal transduction (GO:0007165)	796	−	2.67E−05		
developmental process (GO:0032502)	607	−	7.18E−05		
regulation of transcription from RNA polymerase II promoter (GO:0006357)	245	−	9.40E−05		
cell communication (GO:0007154)	881	−	1.08E−04		
multicellular organismal process (GO:0032501)	451	−	5.14E−03		
intracellular signal transduction (GO:0035556)	388	−	5.44E−03		
single-multicellular organism process (GO:0044707)	450	−	5.58E−03		
nervous system development (GO:0007399)	198	−	8.09E−03		
cell differentiation (GO:0030154)	133	−	8.68E−03		
nucleobase-containing compound metabolic process (GO:0006139)	1160	−	1.40E−02		
steroid metabolic process (GO:0008202)	47	+	1.56E−02		
cellular component movement (GO:0006928)	151	−	3.72E−02		
system development (GO:0048731)	326	−	4.79E−02		
response to stimulus (GO:0050896)	787			+	1.08E−02
immune response (GO:0006955)	161			+	1.19E−02

^a^ +/− Overrepresentation or underrepresentation.

^b^ Bonferroni-corrected *p* value.

Table 8 shows the KEGG pathway enrichments for the global gene expression plasticity in the thyroids of BS and RIR chickens. One and two pathways were enriched in Cold/Warm_BS and Cold/Warm_RIR, respectively, all of which were down-regulated. The “mRNA surveillance pathway” is a quality control mechanism that detects and degrades abnormal mRNAs [76]. Down-regulation of this pathway in Cold/Warm_BS suggests the negative effects of cold on the expression of related genes. The “ribosome” pathway was down-regulated in Cold/Warm_RIR, in accord with the observation that the “ribosome” was more highly expressed in RIR than in BS birds under warm, but not under cold, conditions (Table 6). Ribosomes are directly associated with gene expression, the down-regulation of the “ribosome” pathway in RIR chickens under cold stress also indicated some negative effects of cold on the expression of related genes. The “steroid biosynthesis” pathway, which is part of the “lipid metabolism” pathway and plays an important role in maintaining cellular membrane structure and function [77, 78], was also down-regulated in cold-exposed RIR birds.

**Table 8:** GSEA tests for KEGG pathway enrichments for gene expression plasticity in BS and RIR chicken breeds.

Gene set	Description	Cold/Warm_BS			Cold/Warm_RIR		
		ES	NES	FDR	ES	NES	FDR^a^
gga03015	mRNA surveillance pathway	−0.237	−2.293	0.020			
gga00100	steroid biosynthesis				−0.652	−3.065	0.000
gga03010	ribosome				−0.180	−2.234	0.008

^a^ BH-adjusted *p* value.

Although most of the enriched categories and pathways tended to be down-regulated in BS chickens under cold conditions (Tables 7 and 8), “immune response” and “DNA replication” were still highly expressed in BS compared with RIR thyroids (Table 5). These results likely reflect the higher activity of the thyroid in BS chickens in the related processes, which may be related to the adaptation to the harsh conditions in its place of origin. “Immune response” was up-regulated in Cold/Warm_RIR (Table 7) as well as in BS/RIR_Cold and BS/RIR_Warm (Table 5), indicating the potential influences of the process on thyroid gland functions. Notably, no common categories or pathways were shared between BS and RIR thyroids in response to cold, reflecting different response strategies in these two breeds.

With regard to the expression of the 12 genes related to TH biosynthesis and release, there was no significant differential expression between environments in either breed (S6 File), suggesting that serum TH levels are not merely determined by the thyroid. As stated before, thyroid function is directly affected by the activity of the upstream organs (hypothalamus and pituitary), and ultimately influences serum hormone levels [70], while any processes during hormone biosynthesis, storage, release, transportation, and metabolism can also affect serum hormone levels [71–74]. Our current results may indicate that the differences in TH levels are caused by the TH degradation pathways, though further studies are needed to investigate this issue. More research also needs to focus on the upstream organs, to deepen our understanding of the mechanisms regulating serum hormone levels, and to identify the potential mechanisms underlying cold adaptation. Furthermore, linking the whole-animal performance to transcriptional plasticity in multiple key organs will allow the optimal thermal window and the negative effects of suboptimal conditions to be characterized at the molecular level, thereby shedding light on the molecular mechanisms underpinning the observed physiological traits.

Chickens and other animals have the ability to modify their behavior and physiology to cope with environmental challenges, based on the costs and benefits; however, most breeding programs in recent decades have mainly targeted economic traits such as reproduction, egg quality, growth rate, and feed efficiency, whilst generally ignoring fitness-related traits, leading to reduced adaptability. Highly performing lines have been widely reported to be characterized by low adaptability, decreased performance, and higher sensitivity under suboptimal conditions compared with their wild counterparts or other, less-intensively selected lines [25, 26, 79–82]. RIR generally have higher egg production performance than BS chickens under optimal conditions, determined by the breed’s genomic features shaped by artificial selection. However, egg production performance and body weight decreased significantly in RIR birds following long-term cold exposure in the present study, which, together with a lower responsiveness of the thyroid transcriptome, may indicate that artificial selection in this breed has mainly focused on production performance in the past, potentially reducing its adaptability. As a breed characterized by high laying performance and high meat yield, the RIR is probably the best known chicken breed in the USA, and possibly the world. However, the results of this study suggest that future breeding programs should consider maintaining the adaptability of RIRs, as well as of other farm animals.

### Differential splicing

Alternative splicing is one of the main mechanisms that forge transcriptome plasticity. It can lead to multiple isoforms of a given gene, and can thus affect the functions, binding sites, stability, and efficiency of the proteins [83, 84]. Abundant evidence indicates that alternative splicing is important for adaptive responses to various environmental stressors [85–88]. Differential splicing analysis detects the differences in alternative splice-site usage between compared samples, but is irrelevant to differential gene expression. Analyzing differential splicing will thus help to decipher regulatory mechanisms that are missed by differential gene expression analysis. We used the Bioconductor package DEXSeq, which implements a method to test for differential exon usage in comparative RNA-Seq studies, to detect differential splicing events in the present analysis [41].

**Fig 6:**
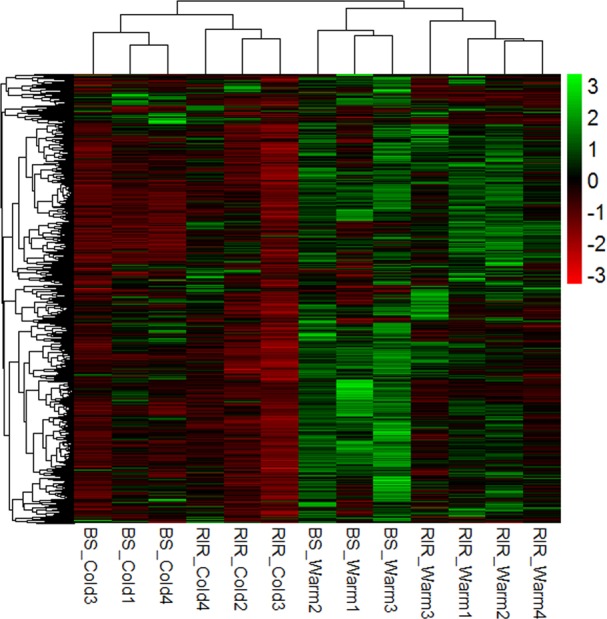
Heatmap of differentially spliced genes.

We analyzed a total of 220,717 exons, and 10,053 differential splicing events were detected with BH-adjusted *p* values < 0.05, corresponding to 3759 genes (S7 File), or about 27.89% of the total detected genes and 15.11% of the total annotated genes in the reference genome. Further hierarchical cluster analysis of the spliced genes showed discernible patterns among the breed and environment combinations (Fig 6). GO and KEGG enrichments of the differentially spliced genes are shown in Tables 9 and 10, respectively. Many enrichments were related to RNA splicing and processing, and gene expression, e.g., “RNA splicing, via transesterification reactions”, “mRNA splicing, via spliceosome”, “mRNA processing”, “RNA metabolic process”, “translation”, “ribosome”, “spliceosome”, “RNA transport”, and “protein processing in endoplasmic reticulum”. Since the genes in these GO categories and KEGG pathways are responsible for regulating splicing and expression of other genes, the differential splicing in these genes may provide a high-order regulatory mode and generate comprehensive influences on overall splicing and gene expression. For example, levels of or mutations in some of the core splicing components of the spliceosome can affect splicing in a transcript-specific manner, as shown in yeast [89, 90] and *Drosophila* [91], and in a tissue-specific manner in mouse [92]. Rapid transcript-specific changes in splicing of ribosomal protein-encoding genes in response to environmental stress have also been reported in *Saccharomyces cerevisiae* [93]. The present results also suggest that splicing regulatory factors may modulate the alternative splicing of their own pre-mRNAs by autoregulation and cross-regulation, which have been widely investigated by the literature [94, 95].

**Table 9:** GO overrepresentation analysis of differentially spliced genes.

Biological Process	#Genes	+/−^a^	*p* value^b^
RNA splicing, via transesterification reactions (GO:0000375)	34	+	4.06E−03
mRNA splicing, via spliceosome (GO:0000398)	42	+	1.22E−03
mRNA processing (GO:0006397)	54	+	2.53E−04
translation (GO:0006412)	61	+	1.67E−04
protein localization (GO:0008104)	60	+	4.55E−02
intracellular protein transport (GO:0006886)	159	+	2.01E−06
protein transport (GO:0015031)	163	+	1.62E−06
vesicle-mediated transport (GO:0016192)	126	+	4.59E−04
cellular component biogenesis (GO:0044085)	91	+	1.43E−02
RNA metabolic process (GO:0016070)	151	+	3.68E−03
catabolic process (GO:0009056)	141	+	1.15E−02
cellular component organization or biogenesis (GO:0071840)	240	+	1.46E−04
protein metabolic process (GO:0019538)	273	+	5.24E−05
cellular component organization (GO:0016043)	209	+	1.97E−03
transport (GO:0006810)	256	+	9.83E−04
localization (GO:0051179)	280	+	5.60E−04
primary metabolic process (GO:0044238)	687	+	1.10E−07
metabolic process (GO:0008152)	795	+	1.03E−07
cellular process (GO:0009987)	902	+	8.18E−03

^a^ +/− Overrepresentation or underrepresentation.

^b^ Bonferroni-corrected *p* value.

**Table 10:** KEGG overrepresentation analysis of differentially spliced genes.

Gene set	Description	#Gene	*p* value	FDR^a^
gga03010	ribosome	77	3.55E−15	5.40E−13
gga04141	protein processing in endoplasmic reticulum	77	4.26E−07	3.24E−05
gga00020	citrate cycle (TCA cycle)	19	1.64E−05	8.33E−04
gga04510	focal adhesion	77	1.12E−04	4.25E−03
gga01200	carbon metabolism	48	2.03E−04	6.17E−03
gga03050	proteasome	22	4.42E−04	1.12E−02
gga00010	glycolysis / Gluconeogenesis	25	1.56E−03	3.39E−02
gga00500	starch and sucrose metabolism	15	2.08E−03	3.84E−02
gga01230	biosynthesis of amino acids	29	2.28E−03	3.84E−02
gga04520	adherens junction	33	2.87E−03	4.18E−02
gga03013	RNA transport	57	3.02E−03	4.18E−02
gga03040	spliceosome	51	3.45E−03	4.37E−02

^a^ BH-adjusted *p* value.

**Fig 7:**
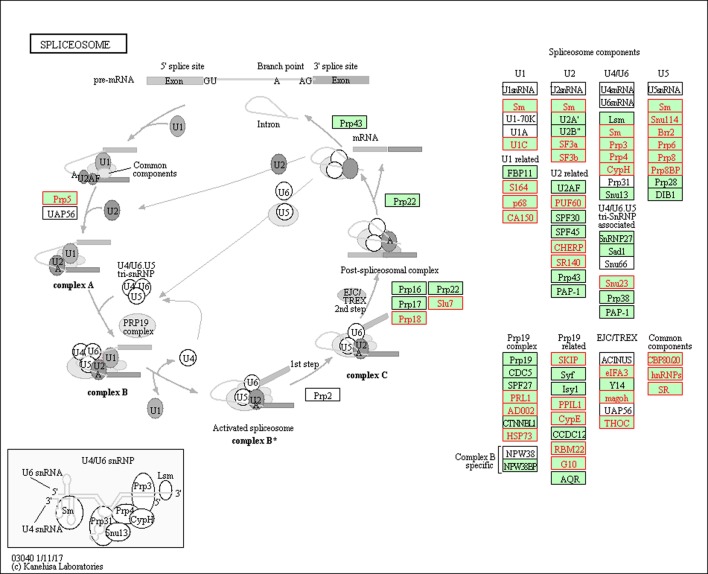
Differentially spliced genes (colored red) associated with the spliceosome pathway.

The spliceosome is a large ribonucleoprotein (RNP) complex including the U1, U2, U4/6, and U5 small nuclear ribonucleoproteins (snRNPs), which carries out the removal of introns from pre-mRNAs [96]. We detected many differential splicing events within the components of the spliceosome, such as the U2-specific splicing factors SF3a and SF3b (Fig 7 and S7 File), which are involved in stabilizing the U2/BS (branch site) duplex [97] and members of the DExD/H-box family of RNA unwindases/RNPases, driving the rearrangements of RNA–RNA and RNA–protein interactions required for splicing [97, 98]. Saltzman et al. [95] reported that the spliced transcript of the core spliceosomal machinery (SmB/B′) through the inclusion of a highly conserved PTC-introducing exon played important roles in homeostatic autoregulation and controlled the expression levels of other mRNAs by nonsense-mediated mRNA decay (NMD).

**Fig 8:**
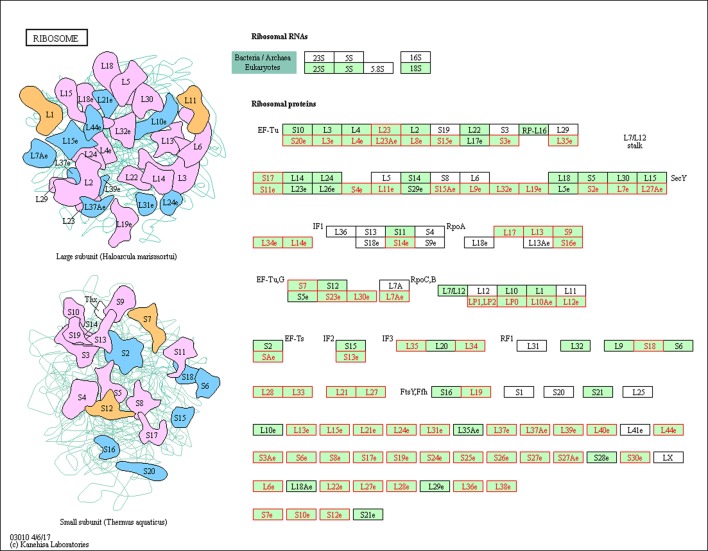
Differentially spliced ribosomal genes (colored red).

**Fig 9:**
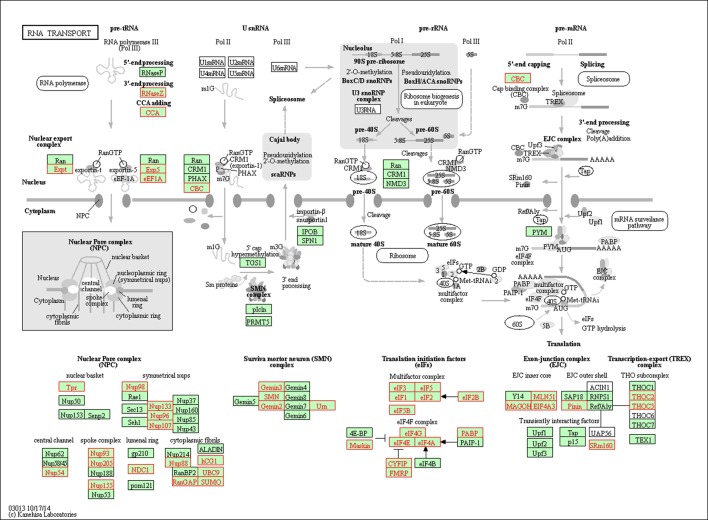
Differentially spliced genes (colored red) associated with the RNA transport pathway.

The ribosome is responsible for translating the genetic information of the mRNA into protein. It comprises two subunits consisting of multiple proteins and RNAs. We identified numerous differential splicing events within the ribosome components (Fig 8). Among the identified spliced ribosomal genes, *RPL3* (ribosomal protein L3) has been reported to carry out autoregulation in splicing in humans, resulting in a PTC-introducing transcript that is further degraded by NMD, leading to regulation of ribosomal protein expression levels [99]. A similar regulatory mechanism was reported for *RPL12* (ribosomal protein L12) in *Caenorhabditis elegans* [100]. RNA transport from the nucleus to the cytoplasm is fundamental for gene expression. We found that the RNA transport pathway was significantly enriched (Table 10 and Fig 9) and the differentially spliced genes mainly affected the nuclear pore complex (NPC), such as multiple nucleoporins (Nups) and translation initiation factors (eIFs). The NPC acts as the gateway between the nucleus and the cytoplasm, and regulates the nucleocytoplasmic trafficking of RNAs and other macromolecules. Nups play a crucial role in gene expression regulation, DNA replication, genome integrity maintenance, and chromosome segregation [101], while eIFs are involved in the initiation phase of eukaryotic translation, and directly regulate protein synthesis. Other representative GO categories and KEGG pathways enriched by the differentially spliced genes included “protein transport”, “vesicle-mediated transport”, “focal adhesion”, “adherens junction”, and “cellular component organization or biogenesis”. Moreover, many categories and pathways involved in metabolic process (e.g. “catabolic process”, “protein metabolic process”, “citrate cycle”, “carbon metabolism”, and “proteasome”) were also significantly enriched (Tables 9 and 10). Most of these were missed by the differential gene expression analysis, confirming the power of differential splicing analysis to reveal regulatory mechanisms, and suggesting that gene expression and alternative splicing control distinct cellular pathways in the chicken thyroid.

**Fig 10:**
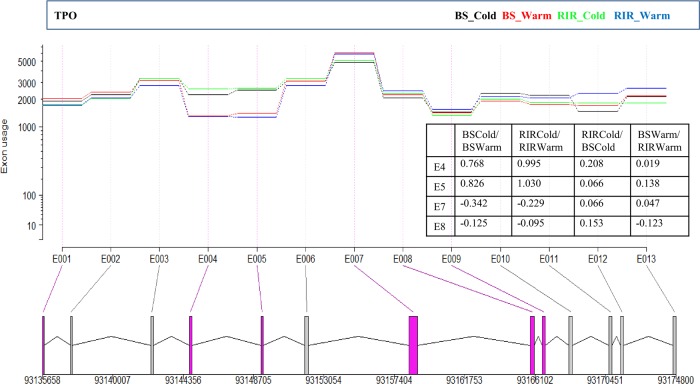
Differential splicing events within *TPO*. Exons with FDR < 0.05 are colored red (bottom panel). The table in the right panel shows the exon usage log2-fold changes in each comparison.

We investigated the differential splicing events of the 12 genes involved in TH synthesis and release (S6 File). After filtering the 3,759 identified differentially spliced genes (S7 File) using the threshold of FDR < 0.05 and exon usage log2FC ≥ 1, one gene, *TPO* (thyroid peroxidase), was retained (Fig 10). TPO oxidizes iodide ions to iodine atoms for the production of THs. Notably, the chicken *TPO* gene has only one transcript annotated in the Ensembl reference Gallus_gallus-5.0 (S7 Fig), while human *TPO* has 15 transcripts annotated in the Ensembl reference GRCh38, indicating the need to improve the chicken reference genome. Exons 4 and 5 showed usage log2-fold changes of about 1 between RIR_Cold and RIR_Warm, and usage log2-fold changes of around 0.8 between BS_Cold and BS_Warm, (Fig 10). Moreover, *TPO* was highly expressed with FPKM of about 592–1,754 in the respective groups, thus supporting the detected differential exon usage. Cold temperature appeared to impact on *TPO* splicing similarly in BS and RIR chickens. The depletion of exons 4 and 5 resulted in a transcript containing multiple open reading frames, though the functions of the spliced transcript require further investigation.

In the present study, we profiled the thyroid transcriptomes of BS and RIR chickens under cold and warm environments, and analyzed the breed differences and transcriptional plasticities of the thyroid transcriptomes. The results of the different parts of the study were mutually supportive, thus reinforcing the reliability of the study. Furthermore, we also identified abundant alternative splicing events, thus identifying a valuable alternative splicing repertoire for the chicken thyroid, and facilitating future investigations of the mechanisms regulating thyroid gland functions.

### qPCR

We examined the expression levels of 10 DEGs with qPCR to confirm the results of the RNA-Seq analysis. The sequences of the primers and the qPCR validation results are shown in S2 Table and S8 Fig, respectively. The qPCR and RNA-Seq results were in good agreement, with a correlation coefficient of 0.93.

## Conclusions

In conclusion, the present study demonstrated that BS chickens had higher cold tolerance and performed better under cold conditions. BS chickens showed higher serum thyroid hormone concentrations than RIRs in both environments. Transcriptional plasticity analysis revealed different adaptive responses in BS and RIR thyroids to cope with cold conditions, and showed higher responsiveness in BS compared with RIR chickens. Cold conditions reduced breed differences in the thyroid transcriptome compared with warm conditions. A total of 10,053 differential splicing events in the chicken thyroid were detected, and the mainly affected processes included RNA splicing and processing, and gene expression, identifying a valuable alternative splicing repertoire for the chicken thyroid. Overall, the results of this study provide novel clues for future studies of the molecular mechanisms underlying cold adaptation and/or acclimation in chickens.

## Supporting information

S1 Fig Functional distribution of the top 200 highly expressed genes in BS_Cold. (DOCX)

S2 Fig Functional distribution of the top 200 highly expressed genes in BS_Warm. (DOCX)

S3 Fig Functional distribution of the top 200 highly expressed genes in RIR_Cold. (DOCX)

S4 Fig Functional distribution of the top 200 highly expressed genes in RIR_Warm. (DOCX)

S5 Fig Venn diagram of the top 200 highly expressed genes in each group. (DOCX)

S6 Fig MDS plots for the total 16 samples. (DOCX)

S7 Fig *TPO* transcript annotated in the Ensembl chicken genome. (DOCX)

S8 Fig Results of qPCR validation. (DOCX)

S1 Table Comparisons between the assembled transcripts and reference annotation. (DOCX)

S2 Table Primer sequences. (DOCX)

S1 File Global gene expression in the thyroid. (XLSX)

S2 File DEGs for BS vs. RIR under warm environment. (XLSX)

S3 File DEGs for BS vs. RIR under natural cold environment. (XLSX)

S4 File DEGs between cold and warm environments in BS chickens. (XLSX)

S5 File DEGs between cold and warm environments in RIR chickens. (XLSX)

S6 File Expression levels of thyroid-related genes. (XLSX)

S7 File Alternative splicing analysis. (XLSX)
