# Supplementary figures and images for: Thyroid transcriptome analysis reveals different adaptive responses to cold environmental conditions between two chicken breeds
Source: PLoS One. 2018 Jan 10;13(1):e0191096. doi: 10.1371/journal.pone.0191096 (PMC5761956; doi:10.1371/journal.pone.0191096)

Fig S1. Functional distribution for the top 200 highly expressed genes of BS_Cold.

**
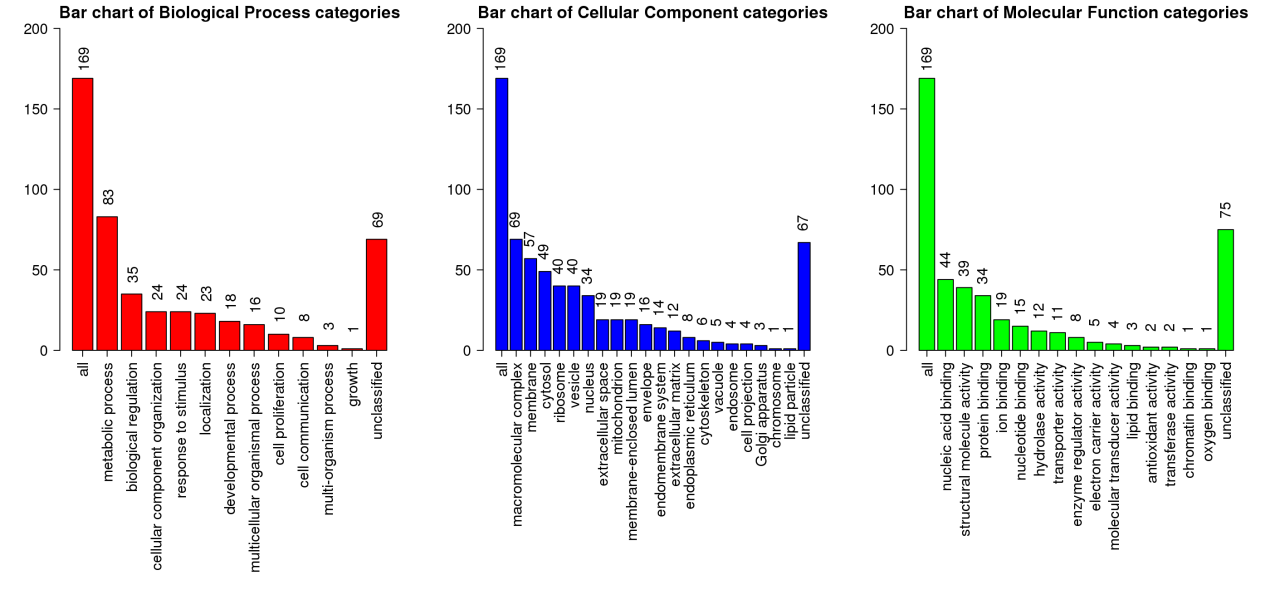
**

Supplement: S1 Fig — (DOCX) [file pone.0191096.s001.docx]

Fig S2. Functional distribution for the top 200 highly expressed genes of BS_Warm.

**
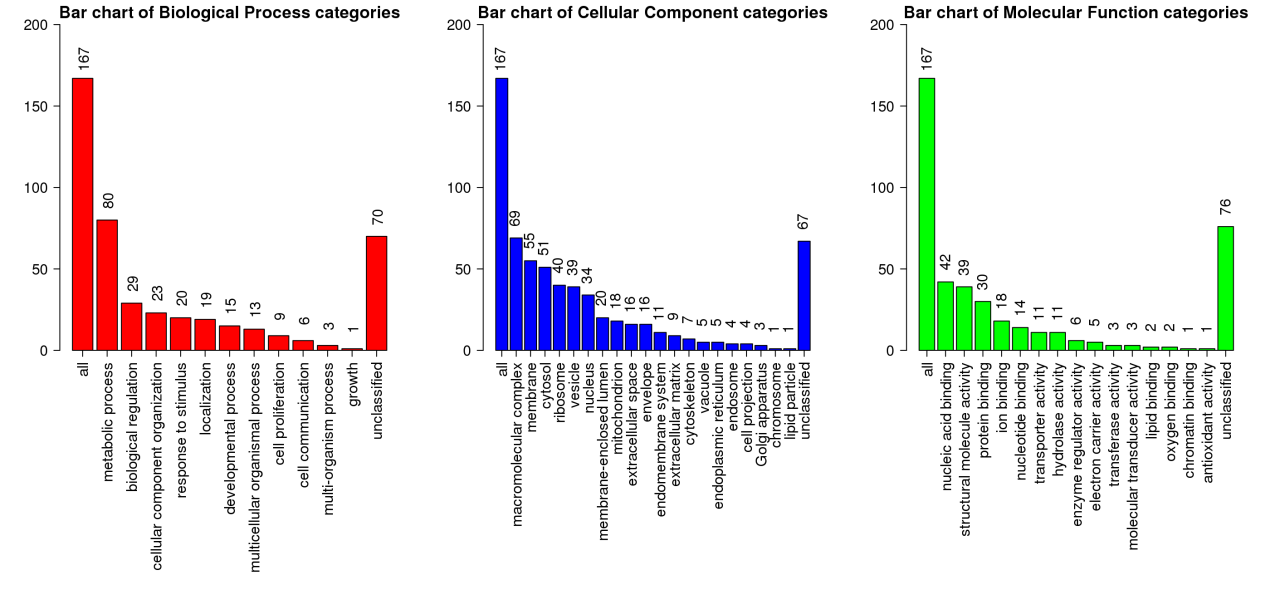
**

Supplement: S2 Fig — (DOCX) [file pone.0191096.s002.docx]

Fig S3. Functional distribution for the top 200 highly expressed genes of RIR_Cold.

**
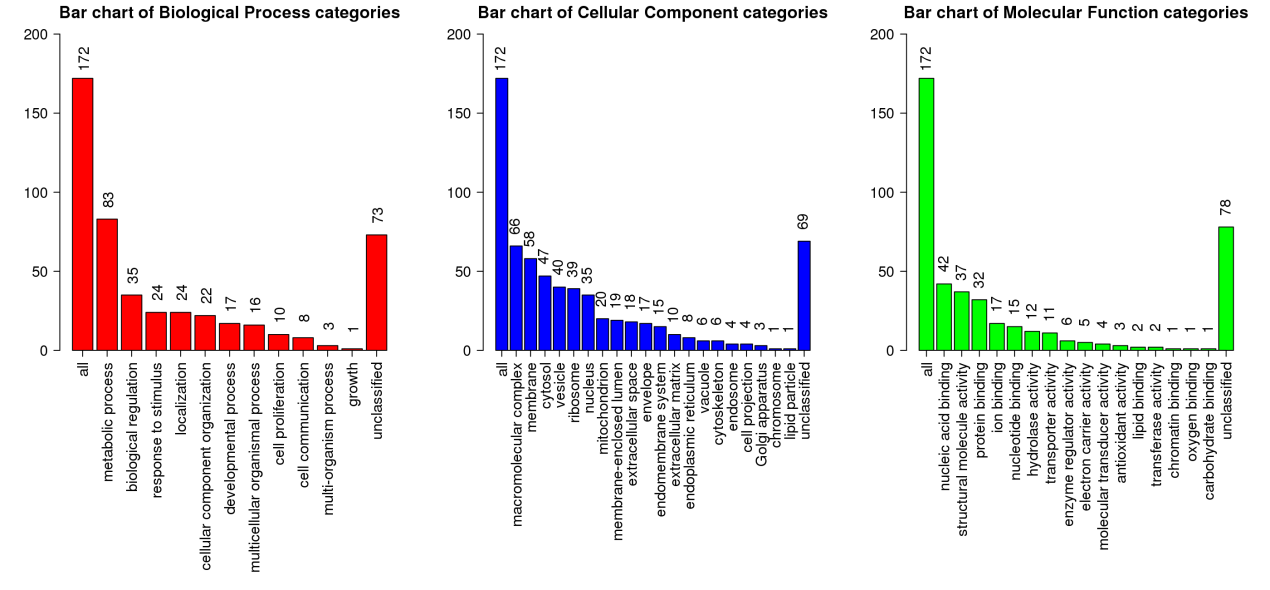
**

Supplement: S3 Fig — (DOCX) [file pone.0191096.s003.docx]

Fig S4. Functional distribution for the top 200 highly expressed genes of RIR_Warm.

**
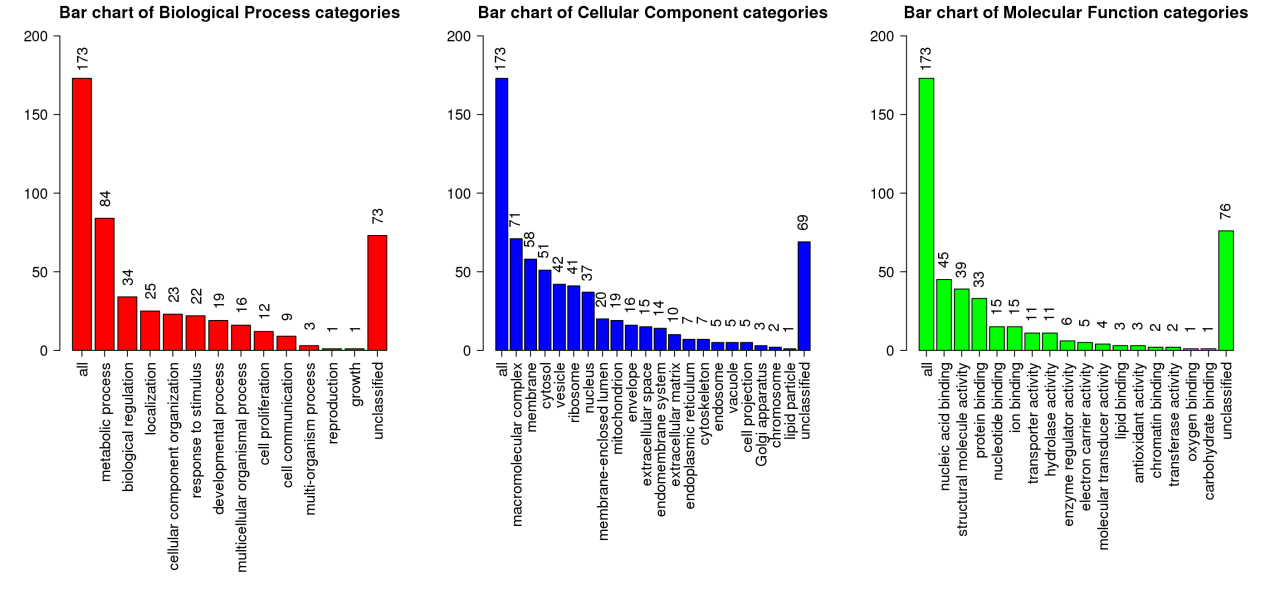
**

Supplement: S4 Fig — (DOCX) [file pone.0191096.s004.docx]

**
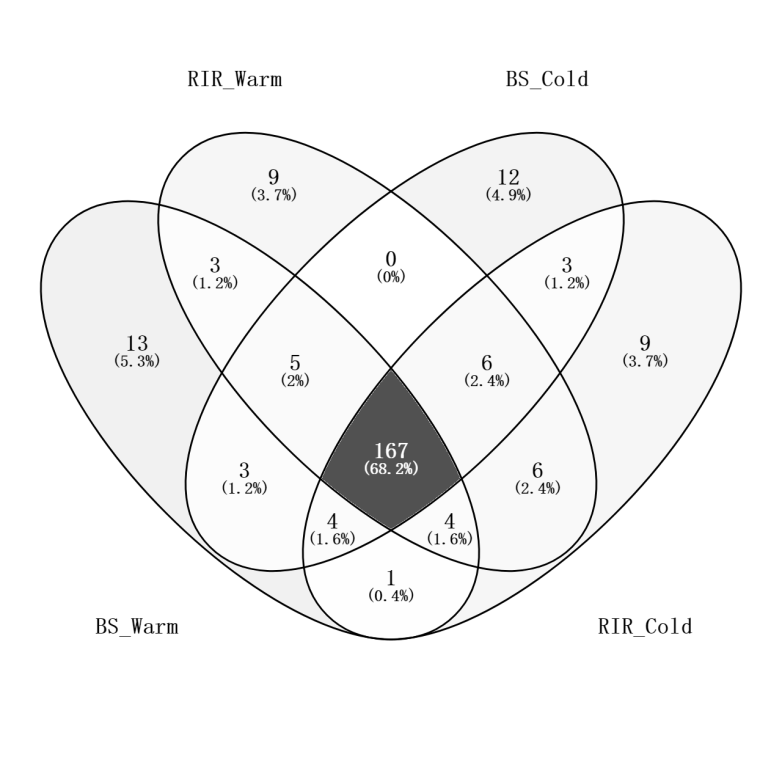
**

Fig S5. Venn diagram for the top 200 highly expressed genes in each group.

Supplement: S5 Fig — (DOCX) [file pone.0191096.s005.docx]

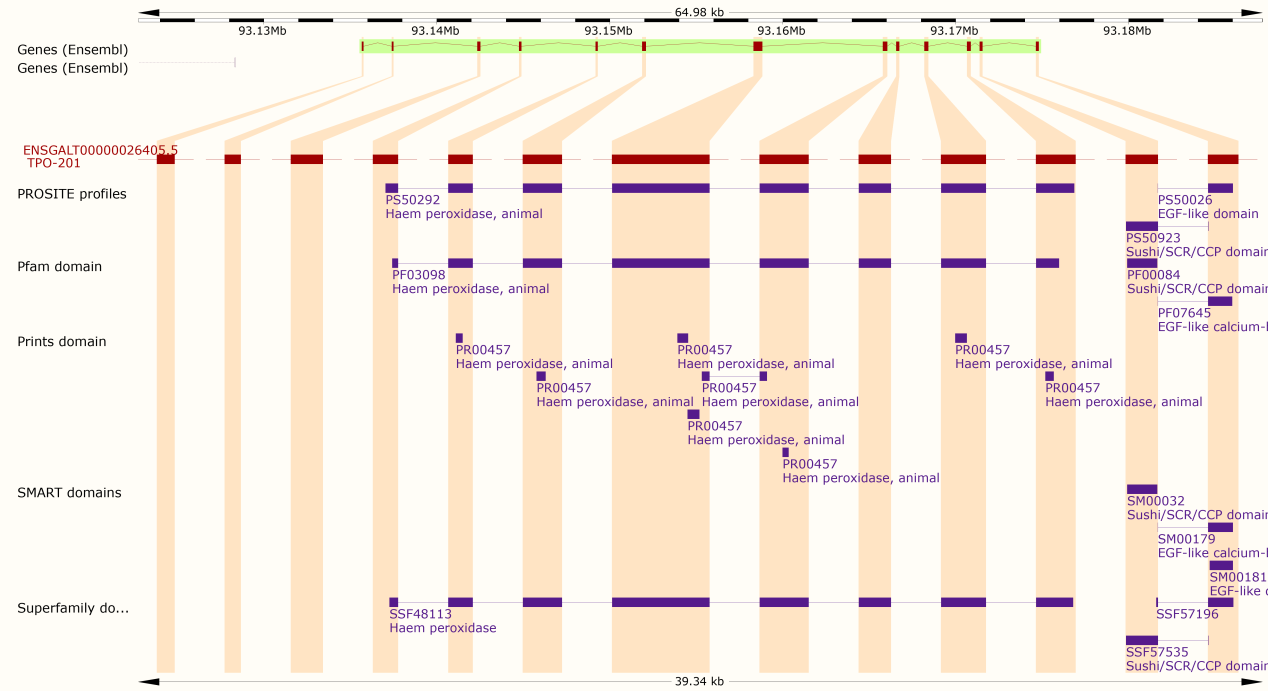


Fig S7. TPO transcript annotated in the Ensembl chicken genome Gallus_gallus-5.0.

Supplement: S7 Fig — (DOCX) [file pone.0191096.s007.docx]

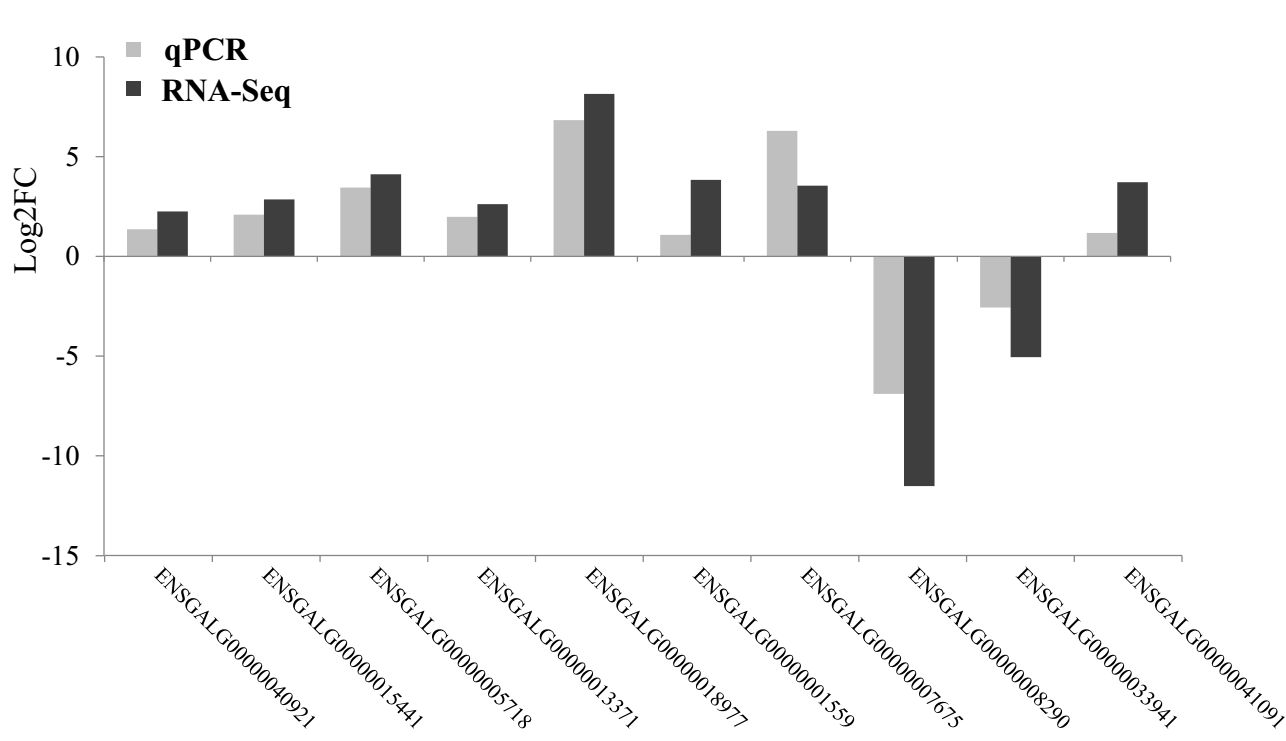


**Figure S8. Results of qPCR validation.**

Supplement: S8 Fig — (DOCX) [file pone.0191096.s008.docx]
