# Supplementary material for: Thyroid transcriptome analysis reveals different adaptive responses to cold environmental conditions between two chicken breeds
Source: PLoS One. 2018 Jan 10;13(1):e0191096. doi: 10.1371/journal.pone.0191096 (PMC5761956; doi:10.1371/journal.pone.0191096)
Supplement: S6 Fig — (DOCX) [file pone.0191096.s006.docx]

**
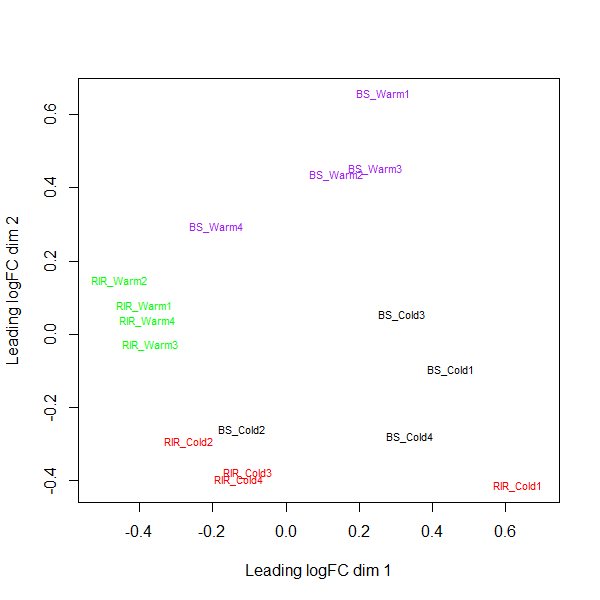
**

Fig S6. **Multidimensional scaling (MDS)** plots. BS_Cold2, RIR_Cold1, and BS_Warm4 were recognized as outliers.
