# Supplementary material for: Thyroid transcriptome analysis reveals different adaptive responses to cold environmental conditions between two chicken breeds
Source: PLoS One. 2018 Jan 10;13(1):e0191096. doi: 10.1371/journal.pone.0191096 (PMC5761956; doi:10.1371/journal.pone.0191096)
Supplement: S1 Table — (DOCX) [file pone.0191096.s009.docx]

**Table S1. Assembly of each group compared to the reference annotation.**

| Class code^a^ | BSCold | | RIRCold | | RIRWarm | | BSWarm | |
| --- | --- | --- | --- | --- | --- | --- | --- | --- |
|  | N | % | N | % | N | % | N | % |
| u | 7024 | 11.00 | 6546 | 10.52 | 5112 | 8.52 | 5554 | 8.83 |
| i | 1294 | 2.03 | 1166 | 1.87 | 724 | 1.21 | 877 | 1.39 |
| j | 14250 | 22.32 | 13475 | 21.66 | 13645 | 22.75 | 15509 | 24.66 |
| x | 1083 | 1.70 | 893 | 1.44 | 845 | 1.41 | 1068 | 1.70 |
| y | 119 | 0.19 | 115 | 0.18 | 111 | 0.19 | 120 | 0.19 |
| o | 763 | 1.20 | 674 | 1.08 | 631 | 1.05 | 740 | 1.18 |
| p | 1351 | 2.12 | 1373 | 2.21 | 964 | 1.61 | 1045 | 1.66 |
| = | 37663 | 58.99 | 37663 | 60.55 | 37663 | 62.80 | 37663 | 59.90 |
| e | 274 | 0.43 | 281 | 0.45 | 239 | 0.40 | 272 | 0.43 |
| s | 21 | 0.03 | 17 | 0.03 | 35 | 0.06 | 32 | 0.05 |
| Total | 63842 | 100.00 | 62203 | 100.00 | 59969 | 1.00 | 62880 | 100.00 |

^a^ Class code definition; u: unknown, intergenic transcript; i: transcript falls entirely within a reference intron; j: new isoform; x: exonic overlaps with reference on the opposite strand; y: rare non-exonic overlaps; o: generic exonic overlap with a reference transcript; p: possible polymerase run-on fragment; =: transcript has exactly the same introns as the reference transcript; e: possible pre-mRNA fragment; s: intron of predicted transcript overlaps a reference intron on the opposite strand.
