# Supplementary material for: Thyroid transcriptome analysis reveals different adaptive responses to cold environmental conditions between two chicken breeds
Source: PLoS One. 2018 Jan 10;13(1):e0191096. doi: 10.1371/journal.pone.0191096 (PMC5761956; doi:10.1371/journal.pone.0191096)
Supplement: S2 Table — (DOCX) [file pone.0191096.s010.docx]

**Table S2. Primer sequences.**

| **Gene** | **Forward primer** | **Reverse primer** |
| --- | --- | --- |
|  |  |  |
| ENSGALG00000040921 | AATGAAAACTTGGTGGCACG | GCTTTCTTCTTGACACAAGGC |
| ENSGALG00000015441 | AGCTCCCACTTACAGATGCT | TATACACATCGTCCTGGCCC |
| ENSGALG00000005718 | AAGAAACAAAACCCAGCCCC | AACTTCCTCCCTCCCACAAG |
| ENSGALG00000013371 | CCTTCTCCCTTCCTGCTCAA | CGGAGAAGTTGTAGTGCTGC |
| ENSGALG00000018977 | AGGGCAGTCTTCCAGGAAAG | GAGTAATTCAGCACGGCGTT |
| ENSGALG00000001559 | TACCGCACGCTCATCAACTA | CTCACGTTCTCCCTCCTGG |
| ENSGALG00000008290 | CCTTGCAGGTCACGTCCA | GCTCTCTTTGGCGCTGTAAA |
| ENSGALG00000033941 | ACAGACCATCCCACATCGAG | AAAACCCGCTCACCTTGC |
| ENSGALG00000041091 | GATGGATCAACAACCGCCTC | AGTTGGGGTGAAGGTGTAGG |
| ENSGALG00000007675 | TCAGAGACCTATGACTTGAGCC | GCCAACCACAGATGACGATG |
| ENSGALG00000014442 | AAAGTCCAAGTGGTGGCCATC | TTTCCCGTTCTCAGCCTTGAC |
